# Effect of Lymph Node Count on Pathological Stage III Rectal Cancer with Preoperative Radiotherapy

**DOI:** 10.1038/srep16990

**Published:** 2015-11-19

**Authors:** Qingguo Li, Lei Liang, Lu Gan, Guoxiang Cai, Xinxiang Li, Sanjun Cai

**Affiliations:** 1 Department of Colorectal Surgery, Fudan University Shanghai Cancer Center, Shanghai 200032, China; 2 Department of Oncology, Shanghai Medical College, Fudan University, Shanghai 200032, China; 3 Department of Medical Oncology, Fudan University Shanghai Cancer Center, Shanghai 200032, China

## Abstract

Lymph node (LN) status after surgery for rectal cancer is affected by preoperative radiotherapy. The purpose of this study was to perform a population-based evaluation of the impact of pathologic LN status after neoadjuvant radiotherapy on survival. A total of 1,650 patients receiving neoadjuvant chemotherapy in Surveillance, Epidemiology, and End Results Program (SEER)-registered ypIII stage rectal cancer was analyzed. We identified the optimal cutoff for retrieved LNs as 10 (χ2 = 14.006, *P* < *0.001*), which was validated as an independent prognosis factors in a Cox regression model. Further analysis showed that the LN count was only a prognosis factor with the number from 8 to 16(except for 13).After the number 16, the 5-year survival rate decreased gradually. Collectively, our results confirmed that the number of LNs in yp III stage rectal patients was a prognosis factor only with the numbers from 8 to 16(except for 13). Using the total mesorectal excision technique with an adequate pathologic examination, a large number of LNs retrieved (≥17) might indicate worse tumor response grade and poorer survival.

Preoperative radiotherapy (preop-RT) has become part of standard practice offered to improve treatment outcomes in patients with rectal cancer^1^,^2^. According to the guidelines for colorectal cancer from the National Comprehensive Cancer Network (NCCN), a minimum of twelve lymph nodes (LNs) must be retrieved and examined for accurate staging and the number of metastatic LNs was validated as an independent prognostic factors^3^,^4^,^5^. Many factors can influence the number of LNs harvested after rectal cancer resection and it has been shown that preop-RT might reduce the number of LNs retrieved^6^,^7^. With the decreased LNs retrieval, the prognostic value of the LN count might also diminish. Increasingly, there is doubt regarding the recommended minimum retrieval of 12 LNs for ypTNM staging^8^. There have been some studies of the optimal LNs retrieval cutoff in ypN0 patients, but few studies have focused on ypN(+) patients. One possible method of identifying a reasonable cutoff value that allows the reliable staging of rectal cancer with LNs metastases treated with preop-RT is to analyze patient survival in a large series of patients diagnosed as ypN(+).

The purpose of this study was to assess the impact of the number of LNs examined on survival of rectal cancer patients treated with preop-RT at ypN(+) stage and to determine the optimal number of LNs that should be examined.

## Results

### Patient Characteristics in SEER database

In our 8-year study period, a total of 1,650 with rectal cancer met our selection criteria, including 1,042 male and 608 female. The median patient age was 57 years (IQR 48-66). There were 1,125 patients with ypN1 stage and 525 with ypN2 stage. Patient demographics and pathological features are summarized in Table 1. Patients with ypN2 stage had a lower rate of high/moderate grade tumors, a higher proportion of mucinous/signet ring adenocarcinoma, and higher ratios of ypT3/4 tumor stage, which reached the level of significance (p < 0.001).

### Identification of Cutoff points for the minimum number of LNs retrieved in ypN(+) patients in the SEER database

**Figure 1:**
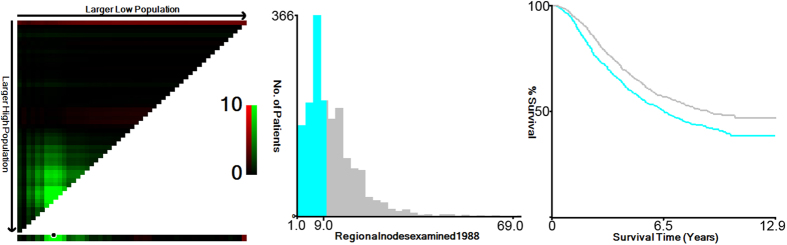
X-tile analysis of survival data from the SEER registry. X-tile analysis was performed using patient data, which were equally divided into training and validation sets, from the SEER registry. X-tile plots of the training sets are shown in the *left panels*, with plots of matched validation sets shown in the *smaller inset*. The optimal cut-point highlighted by the *black circle* in the *left panels* is shown on a histogram of the entire cohort (*middle panels*), and a Kaplan-Meier plot (*right panels*). *P* values were determined using the cutoff point defined in the training set and applying it to the validation set. Figure 1 shows the optimal cutoff point for the ypN (+) patients (10, χ2 = 14.006, *P* < *0.001*).

X-tile plots were constructed and the maximum χ2 log-rank value of 14.006 (P < 0.001) was produced, applying 10 as the optimal cutoff value to divide the cohort into high and low risk subsets in terms of rectal cancer cause-specific survival (RCSS) (Fig. 1). To assess the influence of different LN retrieval on RCSS, we analyzed the individual result using different LN number values ranging from 5 to 19. The number below 5 and over 19 weren’t analyzed to guarantee sufficient statistical power. The 5-year RCSSs were calculated for patients with N (LNs number) or more nodes and less than N nodes (Table 2). The number of LNs retrieved was a prognosis factor only for number ranging from 8 to 16 (except for 13, P value = 0.062).The 5-year RCSS of patients with N or more nodes increased gradually when N ranged from 5 to 9 and the result was roughly the same from 10 to 15 peaking to 65.7% at cutoff 11; but after the number 16, the 5-year RCSS gradually decreased.

### Prognostic value of number of LNs retrieved

The number of LNs and other clinicopathological factors, including elderly patients *(P* < 0.001), black patients (*P* = 0.043), poor and undifferentiated tumor grades (*P* < 0.001), mucinous and signet-ring cancer (*P* = 0.001), advanced TN stages (*P* < 0.001), higher negative to positive LNs ratio (*P* < 0.001), and number of LNs (*P* < 0.001), were significant risk factors for poor survival according to univariate analysis (Table 3). In univariate analysis, age, race, tumor grade, T stage, N stage, higher negative to positive LNs ratio, and LN counts (with an optimal cutoff of 10) were independently and significantly associated with RCSS (Table 3), and a higher number of LNs demonstrated a positive effect on survival (hazard ratio [HR] 0.744; 95% confidence interval [CI] 0.631-0.877, P *<* 0.001) (Table 3).

### Subgroup analysis of the effect of LNs retrieved during ypIII stage

**Figure 2:**
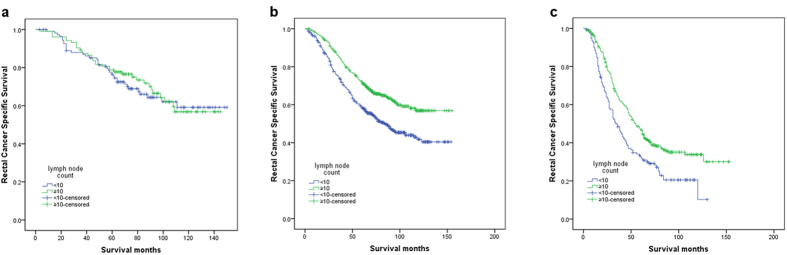
Log-rank tests of rectal cancer cause-specific survival comparing patients with ≥10 lymph nodes with patients who had <10 nodes for (a) stage ypIIIA: χ2 = 0.121, *P* = 0.728; (b) stage ypIIIB: χ2 = 22.817, *P* < 0.001; and (c) stage ypIIIC: χ2 = 13.225, *P* < 0.001.

According to the AJCC-7 rectal cancer staging system, patients with LNs metastases were all classified as ypIII stage, which included three subgroups (i.e., ypIIIA, ypIIIB, and ypIIIC). We then further analyzed these three subgroups. Patients with ypTis (n = 5) and ypTx (n = 23) were excluded from this analysis. The results showed that the retrieved LNs (with a cutoff of 10) were significantly associated with 5-year RCSS, according to the univariate analysis in the ypIIIB (χ2 = 22.817, *P* < 0.001) and ypIIIC subgroups (χ2 = 13.225, *P* *<* 0.001), but not in the ypIIIA subgroup (χ2 = 0.121, *P* = *0.728)* (Fig. 2, Table 4 and 5, [Table t5]). The number of LNs retrieved was also validated as an independent survival factor by multivariate Cox regression in the ypIIIB (LNs≥10, HR 0.698, 95% CI 0.552–0.882, *P* = 0.003) and ypIIIC subgroups (LNs≥10, HR 0.718, 95%CI 0.553–0.933,*P* = 0.013) (Table 4 and 5, [Table t5])

## Discussion

The presence of LN metastases in colorectal cancer is well recognized as one of the most important prognostic factors for long-term outcome^9^,^10^. The total number of LNs retrieved is fundamental in most pathological staging systems for colorectal cancer, including the American Joint Committee on Cancer (AJCC), modified Dukes and Astler and Coller. Inadequate LN evaluation is associated with worse outcomes in terms of tumor recurrence and patient survival, particularly in patients with stage II colorectal cancer^11^,^12^,^13^,^14^. The World Congress of Gastroenterology proposed examining a minimum of 12 LNs to classify stage II colorectal tumors^15^. In the USA, the American Joint Committee on Cancer (AJCC), the American Society of Clinical Oncology (ASCO) and National Comprehensive Cancer Network (NCCN) have also recommended examining at least 12 LNs to assign Stage II rectal disease^16^. However, debate exists regarding the importance of increased LN harvests in stage III colorectal cancer. Le Voyer *et al.* showed that for colon cancer patients with N1 stage, there was an absolute 23% improvement in the 5-year overall survival if >40 LNs were analyzed compared with ≤10 LNs (P < 0.001); and in patients with N2 stage, the 5-year overall survival rates following analysis of >35 and <35 LNs were 71% and 51%, respectively (P = 0.002)^11^. Vather *et al.* reported that the mean numbers of LNs examined in stage III patients who died or were alive within 5 years was 13.1 vs 14.8, respectively, and this difference was statistically significant (P < 0.001)^17^. Chen *et al.* showed that the median survival times for colon cancer patients with 1–7, 8–14 and ≥15 LN retrieval were 46, 52 and 67 months, respectively (P < 0.001)^18^. However, several studies failed to demonstrate a similar association between survival and LNs harvest in stage III disease^13^,^14^,^19^,^20^. Note that our previous study showed that negative LN count, which does not take positive LN into consideration, was an independent prognostic factor for ypIIIB and ypIIIC rectal cancer patients^21^. However, the relationship between LN count and RCSS in ypIII rectal cancer has not been fully investigated. In all present guidelines for rectal cancer clinical practice, total LN count is the main concern. Hence, in this study, we mainly focused on the prognostic significance of the total LNs count in patients with rectal cancer treated with preoperative radiotherapy. We first used X-tile to identify 10 as the optimal cutoff value, and then it was confirmed as one of the optimal cutoff numbers in an additional one-by-one cutoff value analysis from 5 to 19. The 5-year RCSS rates of patients with N (cutoff number) or more nodes gradually increased when N ranged from 5 to 9, which suggested that inadequate LN retrieval in LN positive rectal cancer patients may also reflect the failure to remove the involved LNs, particularly in IIIB and IIIC stage patients in our study, thus increasing the risk of local recurrence and distant metastasis. In addition, it may be a marker of poor quality surgical or pathologic care, both of which may cause poor survival. However, a 10 LN cutoff was not an independent prognostic factor in stage IIIA patients. This finding could be explained by the fact that there was a low percentage(3.3%) of T1-T2 patients with more advanced stage than stage IIIA^22^, therefore, the probability of understaging is significantly low.

The intended purpose of preop-RT is tumor down-staging by decreasing the primary tumor bulk and associated LN metastases. Some authors have demonstrated that neoadjuvant therapy may result in radiation-induced lymphocyte destruction and stromal fibrosis resulting in alterations of the morphology of the LNs, making it difficult to detect them^23^,^24^. Recent research has shown that a decreased number of LNs is related to good tumor response. de Campos-Lobato *et al.* have shown that retrieving fewer than 12 LNs in the proctectomy specimen of rectal cancer patients treated with preoperative chemoradiation does not affect their overall survival and may be a marker of greater tumor response and, consequently, decreased LR rate^25^. Rullier *et al.* were also unable to identify any relationship between a reduced number of LNs examined and decreased patient survival^26^. Habr-Gama *et al.* have demonstrated good disease-free survival rates in patients with a complete absence of LNs in their specimens^27^. We also found that with a cutoff of less than 7, the number of LNs retrieved was not a prognostic factor. Decreased LN retrieval might reflect improved response to preop-RT rather than inappropriate or suboptimal surgical resection in this setting. Most previously published articles determined LN cutoffs considering only a single value, while they barely used one-by-one analysis. In our study, we found that if the cutoff value was greater than 16, the 5-year RCSS rate in patients with a greater number of LNs (i.e., more than the cutoff) would decrease gradually, losing its prognosis value after the number 17(P > 0.05). Several hypotheses could explain this finding. First, the fact that harvesting a higher total number of LNs may result in higher detection rate of metastatic LNs with resultant upstaging of cancer, - a widely accepted concept in surgery; Second, previous study showed that greater LN retrieval could indicate a worse preop-RT response^25^. On average, 5 more LNs were retrieved from ypN2 stage patients compared with ypN1 stage patients (15 *VS* 10), and a poorer tumor regression score results in shorter RCSS rates^25^,^26^,^27^.

This large population-based study has several potential limitations. First, the SEER database does not include information on the administration of chemotherapy. It is possible that additional or concurrent chemotherapy decreases the number of LNs more than preop-RT alone. Second, individual surgeons, pathologists, and other factors may affect LN harvest, but we cannot adjust for these factors. Finally, SEER database also does not report data on preoperative clinical stage, or tumor regression score, so we cannot analyze the relationship between tumor regression score and LNs retrieval in this study.

In conclusion, our analysis of the SEER database revealed that the number of LNs retrieved was a prognostic factor only for numbers ranging from 8 to 16(except for 13) with a maximum χ2 log-rank value of 14.006 at a cutoff of 10. The good tumor response associated with a reduced number of examined LNs may offset the influence of potential under staging with a cutoff of less than 7. Increased LN retrieval (≥17) could indicate worse preop-RT response and poorer RCSS.

## Methods

### Patient selection in the SEER database

Data from the Surveillance Epidemiology and End Results (SEER) Program of the United States National Cancer Institute, release 2015, were utilized for this study. SEER, a population-based cancer registry, collects cancer incidence and survival data from 18 regional population-based registries covering approximately 26% of the US population. The SEER data contain no identifiers and are publicly available for studies of cancer-based epidemiology and TNM staging of colorectal^22^,^28^, gastric^29^, esophageal cancer^30^ and other cancers.

SEER registry patients eligible for this cohort included those with adenocarcinoma, NOS (8150/3), adenocarcinoma in adenomatous polyps (8210/3), villous adenoma (8261/3) or tubulovillous adenoma (8263/3), mucinous adenocarcinoma (8480/3), signet ring cell carcinoma (8490/3) of the rectum diagnosed from 1998 through 2005 and treated with surgical resection. The inclusive study dates were chosen to allow a known treatment sequence of “radiation prior to surgery”. The patient age was limited to 18 to 80 years old. Patients diagnosed after 2006 were excluded to ensure adequate follow-up times. Additional exclusion criteria were as follows: no LNs examined pathologically, ypN0 patients, with synchronous distance metastases, and patients who died within 30 postoperative days.

The following information was retrieved from the SEER database: the patient age, sex, race, extension of primary tumor invasion, radiation sequence, total number of LNs examined, number of involved LNs, grade, survival time, and SEER cause-specific death classification. All cases were restaged based on the AJCC-7 staging. The primary endpoint of the study is RCSS which was calculated from the date of diagnosis to the date of cancer specific death. Deaths attributed to the cancer of interest are treated as events and deaths from other causes are treated as censored observation.

### Statistical analysis

The LNs cutoff points were analyzed using the X-tile program (http://www.tissuearray.org/rimmlab/), which identified the cutoff with the minimum P values from log-rank **χ**^**2**^ statistics for the categorical LNs in terms of survival^31^. The relationship between various clinical and histological variables and survival was evaluated using the Kaplan-Meier method. Differences between survival curves were tested for statistical significance by using log rank test. The Cox proportional hazard regression model was used to identify the variables that could independently influence survival in preop-RT rectal cancer patients. The Mann-Whitney *U* test was used for continuous variables, and the chi-square test was used for categorical variables. The 5-year RCSS rate was estimated from Kaplan-Meier curves. Deaths attributed to the rectal cancer of interest are treated as events and deaths from other causes are treated as censored observation. Statistical analyses were performed with the statistical software package SPSS (Statistical Package for the Social Sciences) for Windows, version 17 (SPSS Inc, Chicago, IL, USA). Two-sided p values of less than 0.05 were considered to be statistically significant.

## Additional Information

**How to cite this article**: Li, Q. *et al.* Effect of Lymph Node Count on Pathological Stage III Rectal Cancer with Preoperative Radiotherapy. *Sci. Rep.* **5**, 16990; doi: 10.1038/srep16990 (2015).

## Figures and Tables

**Table 1:** Characteristics of patients included in the study and make comparison between the ypN1 and ypN2 groups.

Characteristic	Total	ypN1	ypN2	P value
	(n = 1650) (%)	(n = 1125) (%)	(n = 525)	
Media foullow up(mo)	64(2–155)	69 (2–155)	54(2–153)	<0.001
Sex				0.412
male	1042	718(63.8)	324(61.7)	
female	608	407(36.2.)	201 (38.3)	
Age	57(20–80)	58(20-80)	56(21–80)	0.059
Race				0.725
White	1345	921(81.9)	424(80.9)	
Black	128	88(7.8)	40(7.6)	
Other	177	116(10.3)	61(11.6)	
Pathological grading				<0.001
High/Moderate	1133	808(71.8)	325(61.9)	
Poor/Anaplastic	376	216(19.2)	160(30.5)	
Unknown	141	101(9.0)	40(7.6)	
Histotype				<0.001
Adenocarcinoma	1333	955(85.0)	378(72.3)	
Mucinous /Signet ring cell	313	168(15.0)	145(27.7)	
T stage				<0.001
Tis	5	4(0.4)	1(0.2)	
T1/T2	228	182(16.2)	46(8.8)	
T3/4	1393	920(81.9)	473(90.1)	
TX	23	18(1.6)	5(1.0)	
No. of LNs dissected	12(1–69)	10(1–58)	15(4–69)	<0.001

**Table 2:** Univariate analysis of the influence of different total lymph node count on RCSS in ypIII stage rectal cancer patients.

Total lymph node count	No.	5-year CCS	Log-rank χ^2^	P value*
<5	250	58.3%	0.810	0.368
≥5	1400	61.4%		
<6	359	56.1%	3.871	0.049
≥6	1291	62.3%		
<7	457	57.8%	2.008	0.157
≥7	1193	62.1%		
<8	593	55.9%	9.609	0.002
≥8	1057	63.7%		
<9	703	56.6%	11.164	0.001
≥9	947	64.1%		
<10	788	56.6%	14.006	<0.001
≥10	862	64.8%		
<11	902	57.0%	13.492	0.001
≥11	748	65.7%		
<12	991	58.0%	8.515	0.004
≥12	659	65.4%		
<13	1071	59.3%	3.474	0.062
≥13	579	64.0%		
<14	1140	59.4%	4.127	0.042
≥14	510	64.3%		
<15	1192	59.2%	4.771	0.029
≥15	458	65.3%		
<16	1251	59.8%	3.581	0.058
≥16	399	64.4%		
<17	1304	60.4%	2.165	0.141
≥17	346	62.3%		
<18	1355	60.6%	1.058	0.304
≥18	295	62.6%		
<19	1401	60.6%	1.057	0.304
≥19	249	62.7%		

^*^P values refer to the log-rank test of the differences between the two survival curves generated using Kaplan-Meier analysis.

**Table 3:** Univariate and multivariate survival analyses evaluating the number of retrieved LNs influencing RCSS in ypN(+) rectal cancer patients.

Variable		Univariate analysis	Multivariate analysis		
	5-year RCCS	Log rank χ^2^test	P*	HR(95%CI)	P
Sex		0.070	0.791		NI
Male	61.1%				
Female	60.7%				
Age		24.963	<0.001		<0.001
<60	64.6%			Reference	
≥60	55.8%			1.439(1.247–1.659)	
Race		6.311	0.043		0.016
Caucasian	62.1%			Reference	
Black	51.4%			1.404(1.095–1.799)	
others	59.1%			1.170 (0.931–1.471)	
Grade		57.252	<0.001		<0.001
High/ Moderate	65.3%			Reference	
Poor/ Anaplastic	44.7%			1.657(1.412–1.944)	
Unknown	68.5%			0.892(0.672–1.184)	
Histotype		10.876	0.001		0.462
Adenocarcinoma	62.6%			Reference	
Mucinous/signet ring cell	54.5%			1.069(0.895–1.276)	
ypT Stage		22.284	<0.001		0.003
Tis	60.0%			1.173(0.287–4.792)	
T1-2	76..2%			Reference	
T3-4	58.4%			1.565(1.238–1.979)	
Tx	60.3%			1.397(0.722–2.704)	
ypN Stage		81.663	<0.001		<0.001
ypN1	67.5%			Reference	
ypN2	46.7%			1.638(1.358–1.975)	
N/P LNs ratio		90.460	<0.001		0.001
≦2.33	49.9%			Reference	
>2.33	70.6%			0.735(0.631–0.877)	
No. of LNs		14.006	<0.001		<0.001
<10	56.6%			Reference	
≥10	64.8%			0.744(0.631–0.877)	

NI: not included in multivariate survival analysis.

N/P LNs ratio: The ratio of the number of negative lymph nodes to the number of metastatic lymph nodes.

^*^P values refer to the log-rank test of the differences between the two survival curves generated using Kaplan-Meier analysis.

**Table 4:** Univariate and multivariate survival analyses evaluating the number of retrieved LNs influencing RCSS in ypIIIB rectal cancer patients.

Variable	5-year RCCS	Univariate analysis	P*	Multivariate analysis	
		Log rank χ^2^test		HR(95% CI)	P
Sex		0.576	0.448		NI
Male	65.8%				
Female	63.8%				
Age		19.534	<0.001		<0.001
<60	69.3%			Reference	
≥60	59.5%			1.500(1.237–1.820)	
Race		6.580	0.037		0.025
Caucasian	66.1%			Reference	
Black	51.8%			1.556(1.131–2.141)	
others	67.9%			1.025(0.731–1.437)	
Grade		34.685	<0.001		<0.001
High/ Moderate	69.1%			Reference	
Poor/ Anaplastic	47.8%			1.728(1.388-2.151)	
Unknown	74.2%			0.803(0.536–1.203)	
Histotype		5.186	0.023		0.325
Adenocarcinoma	66.5%			Reference	
Mucinous/signet ring cell	58.9%			1.134(0.883–1.455)	
ypT Stage		6.475	0.039		0.009
T1-2	53.8%			Reference	
T3	67.6%			0.973(0.460-2.059)	
T4	60.5%			1.364(0.625–2.975)	
ypN Stage		13.788	<0.001		0.002
ypN1	67.1%			Reference	
ypN2	53.6%			1.616(1.195–2.185)	
N/P LNs ratio		27.218	<0.001		0.070
≦2.33	56.1%			Reference	
>2.33	70.4%			0.801(0.629–1.019)	
No. of LNs		22.817	<0.001		0.003
<10	58.8%			Reference	
≥10	72.0%			0.698(0.552–0.882)	

NI: not included in multivariate survival analysis.

N/P LNs ratio: The ratio of the number of negative lymph nodes to the number of metastatic lymph nodes.

^*^P values refer to the log-rank test of the differences between the two survival curves generated using Kaplan-Meier analysis.

**Table 5 t5:** Univariate and Multivariate survival analyses for evaluating the number of LNs retrieved influencing RCSS in ypIIIC rectal cancer patients.

Variable	5-year CCS	Univariate analysis	P*	Multivariate analysis	
		Log rank χ^2^test		HR(95% CI)	P
Sex		0.716	0.397		NI
Male	40.5%				
Female	45.2%				
Age		7.615	0.006		0.010
<60	45.0%			Reference	
≥60	36.6%			1.383(1.082–1.769)	
Race		1.001	0.606		NI
Caucasian	42.4%				
Black	43.0%				
others	37.6%				
Grade		9.790	0.007		0.018
High/ Moderate	45.2%			Reference	
Poor/Anaplastic	31.4%			1.470(1.126–1.921)	
Unknown	50.4%			1.083(0.709–1.654)	
Histotype		0.149	0.699		NI
Adenocarcinoma	41.5%				
Mucinous/signet ring cell	43.2%				
ypT Stage		4.371	0.037		0.033
T3	50.8%			Reference	
T4	39.2%			1.428(1.030–1.979)	
ypN Stage		0.648	0.421		NI
ypN1	42.4%				
ypN2	41.7%				
N/P LNs ratio		13.621	<0.001		<0.001
≦2.33	37.3%			Reference	
>2.33	56.1%			0.548(0.394–0.762)	
No. of LNs		13.225	<0.001		0.013
<10	32.4%			Reference	
≥10	46.6%			0.718(0.553–0.933)	

NI: not included in multivariate survival analysis.

N/P LNs ratio: The ratio of the number of negative lymph nodes to the number of metastatic lymph nodes.

^*^P values refer to the log-rank test of the differences between the two survival curves generated using Kaplan-Meier analysis.

## References

[b1] Kapiteijn E. *et al.* Preoperative radiotherapy combined with total mesorectal excision for resectable rectal cancer. N Engl J Med 345, 638–646 (2001). 11547717 10.1056/NEJMoa010580

[b2] Sauer R. *et al.* Preoperative versus postoperative chemoradiotherapy for rectal cancer. N Engl J Med 351, 1731–1740 (2004). 15496622 10.1056/NEJMoa040694

[b3] Gunderson L. L., Jessup J. M., Sargent D. J., Greene F. L. & Stewart A. K. Revised TN categorization for colon cancer based on national survival outcomes data. J Clin Oncol 28, 264–271 (2010). 19949014 10.1200/JCO.2009.24.0952 PMC2815715

[b4] Hong K. D., Lee S. I. & Moon H. Y. Lymph node ratio as determined by the 7th edition of the American Joint Committee on Cancer staging system predicts survival in stage III colon cancer. J Surg Oncol 103, 406–410 (2011). 21400524 10.1002/jso.21830

[b5] Suzuki O. *et al.* Number of lymph node metastases is better predictor of prognosis than level of lymph node metastasis in patients with node-positive colon cancer. J Am Coll Surg 202, 732–736 (2006). 16648012 10.1016/j.jamcollsurg.2006.02.007

[b6] Baxter N. N., Morris A. M., Rothenberger D. A. & Tepper J. E. Impact of preoperative radiation for rectal cancer on subsequent lymph node evaluation: a population-based analysis. Int J Radiat Oncol Biol Phys 61, 426–431 (2005). 15667963 10.1016/j.ijrobp.2004.06.259

[b7] Li Q. *et al.* Lymph node count after preoperative radiotherapy is an independently prognostic factor for pathologically lymph node-negative patients with rectal cancer. Medicine 94, e395 (2015). 25621683 10.1097/MD.0000000000000395 PMC4602649

[b8] Leibold T. *et al.* Prognostic implications of the distribution of lymph node metastases in rectal cancer after neoadjuvant chemoradiotherapy. J Clin Oncol 26, 2106–2111 (2008). 18362367 10.1200/JCO.2007.12.7704

[b9] Compton C. C. *et al.* Prognostic factors in colorectal cancer. College of American Pathologists Consensus Statement 1999. Arch Pathol Lab Med 124, 979–994 (2000). 10888773 10.5858/2000-124-0979-PFICC

[b10] Baxter N. N. *et al.* Lymph node evaluation in colorectal cancer patients: a population-based study. J Natl Cancer Inst 97, 219–225 (2005). 15687365 10.1093/jnci/dji020

[b11] Le Voyer T. E. *et al.* Colon cancer survival is associated with increasing number of lymph nodes analyzed: a secondary survey of intergroup trial INT-0089. J Clin Oncol 21, 2912–2919 (2003). 12885809 10.1200/JCO.2003.05.062

[b12] Swanson R. S., Compton C. C., Stewart A. K. & Bland K. I. The prognosis of T3N0 colon cancer is dependent on the number of lymph nodes examined. Ann Surg Oncol 10, 65–71 (2003). 12513963 10.1245/aso.2003.03.058

[b13] Prandi M. *et al.* Prognostic evaluation of stage B colon cancer patients is improved by an adequate lymphadenectomy: results of a secondary analysis of a large scale adjuvant trial. Ann Surg 235, 458–463 (2002). 11923600 10.1097/00000658-200204000-00002 PMC1422459

[b14] Tepper J. E. *et al.* Impact of number of nodes retrieved on outcome in patients with rectal cancer. J Clin Oncol 19, 157–163 (2001). 11134208 10.1200/JCO.2001.19.1.157

[b15] Fielding L. P. *et al.* Clinicopathological staging for colorectal cancer: an International Documentation System (IDS) and an International Comprehensive Anatomical Terminology (ICAT). J Gastroenterol Hepatol 6, 325–344 (1991). 1912440 10.1111/j.1440-1746.1991.tb00867.x

[b16] Klopfleisch R., Weiss A. T. & Gruber A. D. Excavation of a buried treasure–DNA, mRNA, miRNA and protein analysis in formalin fixed, paraffin embedded tissues. Histol Histopathol 26, 797–810 (2011). 21472693 10.14670/HH-26.797

[b17] Vather R. *et al.* Lymph node examination as a predictor of long-term outcome in Dukes B colon cancer. Int J Colorectal Dis 24, 283–288 (2009). 18716784 10.1007/s00384-008-0540-y

[b18] Chen S. L. & Bilchik A. J. More extensive nodal dissection improves survival for stages I to III of colon cancer: a population-based study. Ann Surg 244, 602–610 (2006). 16998369 10.1097/01.sla.0000237655.11717.50 PMC1856560

[b19] Caplin S., Cerottini J. P., Bosman F. T., Constanda M. T. & Givel J. C. For patients with Dukes’ B (TNM Stage II) colorectal carcinoma, examination of six or fewer lymph nodes is related to poor prognosis. Cancer 83, 666–672 (1998). 9708929

[b20] Sarli L. *et al.* Number of lymph nodes examined and prognosis of TNM stage II colorectal cancer. Eur J Cancer 41, 272–279 (2005). 15661553 10.1016/j.ejca.2004.10.010

[b21] Li Q. *et al.* Increased number of negative lymph nodes is associated with improved cancer specific survival in pathological IIIB and IIIC rectal cancer treated with preoperative radiotherapy. Oncotarget 5, 12459–12471 (2014). 25514596 10.18632/oncotarget.2560 PMC4323013

[b22] Gunderson L. L., Jessup J. M., Sargent D. J., Greene F. L. & Stewart A. Revised tumor and node categorization for rectal cancer based on surveillance, epidemiology, and end results and rectal pooled analysis outcomes. J Clin Oncol 28, 256–263 (2010). 19949015 10.1200/JCO.2009.23.9194 PMC2815714

[b23] Fajardo L. F. Effects of ionizing radiation on lymph nodes. A review. Front Radiat Ther Oncol 28, 37–45 (1994). 7982602 10.1159/000423371

[b24] Shvero J. *et al.* Histological changes in the cervical lymph nodes after radiotherapy. Oncol Rep 8, 909–911 (2001). 11410808 10.3892/or.8.4.909

[b25] de Campos-Lobato L. F. *et al.* Less than 12 nodes in the surgical specimen after total mesorectal excision following neoadjuvant chemoradiation: it means more than you think! Ann Surg Oncol 20, 3398–3406 (2013). 23812804 10.1245/s10434-013-3010-x

[b26] Rullier A. *et al.* Lymph nodes after preoperative chemoradiotherapy for rectal carcinoma: number, status, and impact on survival. Am J Surg Pathol 32, 45–50 (2008). 18162769 10.1097/PAS.0b013e3180dc92ab

[b27] Habr-Gama A. *et al.* Absence of lymph nodes in the resected specimen after radical surgery for distal rectal cancer and neoadjuvant chemoradiation therapy: what does it mean? Dis Colon Rectum 51, 277–283 (2008). 18183463 10.1007/s10350-007-9148-5

[b28] Gao P. *et al.* Is the prediction of prognosis not improved by the seventh edition of the TNM classification for colorectal cancer? Analysis of the surveillance, epidemiology, and end results (SEER) database. BMC Cancer 13, 123 (2013). 23496812 10.1186/1471-2407-13-123 PMC3651725

[b29] Wang X. *et al.* Comparison of three lymph node staging schemes for predicting outcome in patients with gastric cancer. Br J Surg 100, 505–514 (2013). 23319421 10.1002/bjs.9014

[b30] Hu Y., Hu C., Zhang H., Ping Y. & Chen L. Q. How does the number of resected lymph nodes influence TNM staging and prognosis for esophageal carcinoma? Ann Surg Oncol 17, 784–790 (2010). 19953333 10.1245/s10434-009-0818-5

[b31] Camp R. L., Dolled-Filhart M. & Rimm D. L. X-tile: a new bio-informatics tool for biomarker assessment and outcome-based cut-point optimization. Clin Cancer Res 10, 7252–7259 (2004). 15534099 10.1158/1078-0432.CCR-04-0713

